# Hydroxysafflor Yellow A protects spinal cords from ischemia/reperfusion injury in rabbits

**DOI:** 10.1186/1471-2202-11-98

**Published:** 2010-08-13

**Authors:** Le-qun Shan, Sai Ma, Xiu-chun Qiu, Yong Zhou, Yong Zhang, Lian-he Zheng, Peng-cheng Ren, Yu-cai Wang, Qing-yu Fan, Bao-an Ma

**Affiliations:** 1 Department of Orthopedic Surgery, Tangdu Hospital, The Fourth Military Medical University, Xi' an 710038, China; 2 Department of Prosthodontics, School of Stomatology, The Fourth Military Medical University, Xi' an 710032, China

## Abstract

**Background:**

Hydroxysafflor Yellow A (HSYA), which is one of the most important active ingredients of the Chinese herb *Carthamus tinctorius L*, is widely used in the treatment of cerebrovascular and cardiovascular diseases. However, the potential protective effect of HSYA in spinal cord ischemia/reperfusion (I/R) injury is still unknown.

**Methods:**

Thirty-nine rabbits were randomly divided into three groups: sham group, I/R group and HSYA group. All animals were sacrificed after neurological evaluation with modified Tarlov criteria at the 48th hour after reperfusion, and the spinal cord segments (L4-6) were harvested for histopathological examination, biochemical analysis and terminal deoxynucleotidyl transferase-mediated dUTP-biotin nick end labeling (TUNEL) staining.

**Results:**

Neurological outcomes in HSYA group were slightly improved compared with those in I/R group. Histopathological analysis revealed that HSYA treatment attenuated I/R induced necrosis in spinal cords. Similarly, alleviated oxidative stress was indicated by decreased malondialdehyde (MDA) level and increased superoxide dismutase (SOD) activity after HSYA treatment. Moreover, as seen from TUNEL results, HSYA also protected neurons from I/R-induced apoptosis in rabbits.

**Conclusions:**

These findings suggest that HSYA may protect spinal cords from I/R injury by alleviating oxidative stress and reducing neuronal apoptosis in rabbits.

## Background

Paraplegia that results from spinal cord ischemia remains a catastrophic complication of thoraco-abdominal aortic surgery. Immediate or delayed paraplegia due to I/R injury of the spinal cord has an incidence of between 4% to 33% [1]. Great efforts, including surgical techniques (such as temporary shunts or partial bypass), pharmacological interventions (such as methylprednisolone), and mechanical methods (such as hypothermia, drainage of cerebrospinal fluid), have been focused on the alleviation of spinal cord ischemia injury [2-4]. Despite all these efforts, no single technique has been proved consistently effective in eliminating I/R-induced neurological dysfunctions. Thus, further investigations are required to find new drugs or techniques to protect against spinal cord I/R injury [5].

Although the exact mechanism of spinal cord I/R injury is not fully understood, oxygen-derived free radicals are widely recognized as an important contributor to neuronal damages [6]. Therefore, antioxidants represent a good candidate for the prevention of neurological deficits related to spinal cord I/R injury.

In traditional oriental medicine, the flower of the safflower plant, namely *Carthamus tinctorius L*, is extensively used for the treatment of cerebrovascular and cardiovascular diseases. HSYA, which is the main chemical component of the safflower yellow pigments, has been demonstrated to have potent antioxidative effect in vitro [7]. Recent studies revealed that HSYA could alleviate I/R injury of the lung, heart [8] and brain [9] via scavenging of free radicals. However, it remains uncertain yet whether HSYA could protect against spinal cord I/R injury.

The purpose of this study was to investigate the protective efficacy of HSYA on the neurological, biochemical and histopathological outcomes of spinal cord I/R injury in rabbits. We hypothesized that HSYA might protect spinal cords from I/R injury via reduction of oxidative stress and inhibition of apoptosis.

## Methods

### HSYA preparation

HSYA powder with 98.0% purity was purchased from Shandong Lvye Natural Medicine Research and Development Center (Shandong, China). HSYA solution was prepared and injected in the form of Ringer's ethyl pyruvate solution at a concentration of 1 mg/ml.

### General animal care and setting of groups

This study was performed in Bone Tumor Institute of Tangdu Hospital, Fourth Military Medical University. All experimental protocols were carried out in accordance with the National Institutes of Health Guide for the Care and Use of Laboratory Animals (NIH publications No. 80-23). In total, 39 adult male New Zealand rabbits, 4 to 5-month old and 2.2 to 2.5-kg body weight, were used and randomly divided into three groups. Animals in sham group (n = 13) were subjected to only laparotomy without aortic occlusion or any pharmacological treatment. The 13 rabbits in HSYA group were given 10.0 mg/kg HSYA through left ear vein 30 min before occlusion and again at the onset of reperfusion. The other 13 rabbits in I/R group underwent similar procedures as animals in HSYA group but received an equivalent dose of saline instead of HSYA.

### Surgery preparation

After an overnight fast with unrestricted access to water, the rabbits were anesthetized with pentobarbital sodium (30 mg/kg, IV) and allowed to breathe spontaneously. Additional doses of pentobarbital sodium were given during the surgery when needed. The left ear vein was cannulated with a 22-gauge catheter to measure the proximal blood pressure and administrate HSYA. Another catheter was inserted into the left femoral artery to measure the distal blood pressure. Blood pressure was monitored continuously by using a calibrated pressure transducer connected to an invasive pressure monitor (Spacelabs Medical Inc., Redmond, WA). During the surgery, rectal temperature was maintained at about 38.5°C by an overhead lamp. Arterial blood was sampled at preischemia, 10 min after ischemia, and 10 min after reperfusion, respectively, for the determination of arterial oxygen tension (PaO2), arterial carbon dioxide tension (PaCO2), pH, and plasma glucose. Arterial blood gases were measured by means of the OMNI Modular System (AVL List GmbH Medizintechnik, Kleiststrabe, Graz, Austria).

### Ischemia-reperfusion procedure

Spinal cord I/R injury models were established according to Savas' description [10]. Animals were placed in supine position. After sterile preparation, a 10 cm midline incision was made to expose the abdominal aorta. Following anticoagulation with 400 unit's heparin, the abdominal aorta was cross-clamped for 20 min using atraumatic vascular clamps at the level of the left renal artery. At the end of the occlusion period, the clamp was removed, and the abdominal wall was subsequently carefully closed in separate layers using 4-0 nylon suture. The animals were placed under an overhead lamp to maintain body temperature until recovery from anesthesia. They were then returned to their home cage, and given prophylactic antibiotic (gentamicin; 40,000 IU) and saline (5 ml, twice a day for 5 days, to prevent dehydration). Bladders were voided manually twice a day until normal function returned.

### Neurological evaluation

Neurological status of the animals was assessed at 24 h and 48 h after reperfusion by an independent observer who had no prior knowledge of the experimental protocol and grouping. Modified Tarlov criteria was used to grade the motor function of hind limbs [11]: grade 0, no movement of the lower limbs; grade 1, minimal movement; grade 2, good movement but unable to stand; grade 3, able to stand and walk but unable to hop normally; and grade 4, normal recovery.

### Histological study

Forty-eight hours after I/R injury, 8 rabbits from each group were reanesthetized with pentobarbital sodium (40 mg/kg). Then intracardiac perfusion was performed with 1000 ml heparinized saline and 500 ml 10% buffered formalin. After perfusion, the lumbar spinal cord (L4-6 segments) was removed immediately, immersed in 10% formaldehyde and stored at 4°C for 48 h. After dehydration in graded ethanol, the spinal cord specimens were embedded in paraffin. Coronal sections of the spinal cord segment were cut at a thickness of 5 μm and stained with hematoxylin and eosin (H&E) for evaluation of structural changes. Injured neurons were identified by intensely eosinophilic cytoplasm, loss of Nissl substance and pyknotic nuclei. The remaining normal neurons in the ischemic ventral spinal cord in each animal, judged by their morphological appearance, were counted in three sections selected randomly from the rostral, middle, and caudal levels of the L5 segment and then averaged. The numbers of normal neurons per section were compared among the three groups.

### Biochemical analysis

Five rabbits from each group were reanesthetized with pentobarbital sodium (40 mg/kg) 48 h after I/R injury. The lumbar spinal cord was immediately removed, washed twice with cold saline solution, placed into glass bottles, labeled and stored at -70°C until processing (maximum 10 h). Spinal cord tissue samples were cut into small pieces, weighed, and homogenized in ice-cold Tris-HCl buffer (50 mmol, pH 7.4). The homogenate was then centrifuged at 5000 g for 30 min to remove debris. For further extraction, the supernatant solution was extracted with an equal volume of an ethanol/chloroform mixture (5/3, volume per volume [v/v]). After the second centrifugation at 5000 g for 60 min, the clear upper layer (the ethanol phase) was taken and used for SOD activity assay. MDA level was determined in the homogenate. Protein concentrations were measured according to Lowry's procedure [12].

Tissue MDA levels were determined by the method described by Wasowicz et al. [13]. Briefly, MDA was reacted with thiobarbituric acid by incubating for 1 h at 95-100°C. After the reaction, fluorescence intensity was measured in the n-butanol phase with a fluorescence spectrophotometry (Hitachi, Model F-4010) (excitation at 525 nm, emission at 547 nm), by comparing with a standard solution of 1,1,3,3 tetraethoxypropane. Results were expressed in terms of nmol/g wet tissue.

The level of total (Cu-Zn and Mn) SOD was studied using the Yi-Sun method, which is based on the inhibition of nitroblue tetrazolium reduction by the xanthine oxidase system as a superoxide generator [14]. Activity was assessed in the ethanol phase of the spinal cord homogenate after ethanol/chloroform mixture was added to the same volume of sample and centrifuged. One unit of SOD was defined as the enzyme amount showing 50% inhibition. SOD activity was expressed as U/mg protein.

### TUNEL staining

TUNEL staining was performed on paraffin sections, using an in situ cell death detection kit (Roche, Germany), in accordance with the manufacture's instructions. Hematoxylin was used to counterstain the sections. A negative control was similarly performed except for replacing TUNEL reaction mixture with label solution (without terminal transferase). Quantitative analysis was performed blindly by counting the number of TUNEL positive neurons in the ventral horns in five microscopic fields. The average number of TUNEL positive neurons in each group was calculated from the five specimens.

### Statistical analysis

All values are presented as mean ± SEM. Statistical analysis was performed with the help of SPSS 12.0. Kruskal-Wallis nonparametric analysis was followed by Mann-Whitney U-test to analyze neurological scores and the numbers of viable motor neurons and apoptotic motor neurons in the anterior spinal cord. MDA content and SOD activity were analyzed with one-way analysis of variance (ANOVA), followed by SNK-q test for multiple comparisons. A *P *value less than 0.05 was considered to be statistically different.

## Results

### Physical parameters

There was no difference between groups at any time point in terms of hemodynamics, rectal temperature, arterial pH, PaCO2, PaO2, and blood glucose concentrations. The distal blood pressure was about 70 to 80 mmHg before blocking the abdominal aorta and it decreased to 10 to 15 mmHg during the period of ischemia. Ten minutes after the beginning of reperfusion, the value of the distal blood pressure was recovered to approximately 90% preischemic level (data not shown).

### Neurological function evaluation

The outcomes of neurological evaluation are presented in Table 1. Animals in sham group displayed no neurological abnormalities at the 24th and 48th hour after reperfusion. The neurological scores in I/R group and HSYA group were significantly lower than that of sham group at both the 24th hour and 48th hour. Animals in HSYA group exhibited better neurological outcomes than I/R group at the 24th and 48th hour after reperfusion.

**Table 1:** The neurological scores of rabbits at 24 h and 48 h after reperfusion (n = 13).

Tarlov Score	Sham Group		I/R Group		HSYA-treatment Group	
			
	24 h	48 h	24 h	48 h	24 h	48 h
0	0	0	0	2	0	0
1	0	0	3	10	1	4
2	0	0	8	1	4	6
3	0	0	2	0	7	3
4	13	13	0	0	1	0
Average Score	4	4	1.92 ± 0.64*	0.92 ± 0.49*	2.62 ± 0.77 *^, #^	1.92 ± 0.76*^, #^

* *P *< 0.05, compared with Sham group. ^# ^*P *< 0.05, compared with I/R group.

### Histopathological study

**Figure 1:**
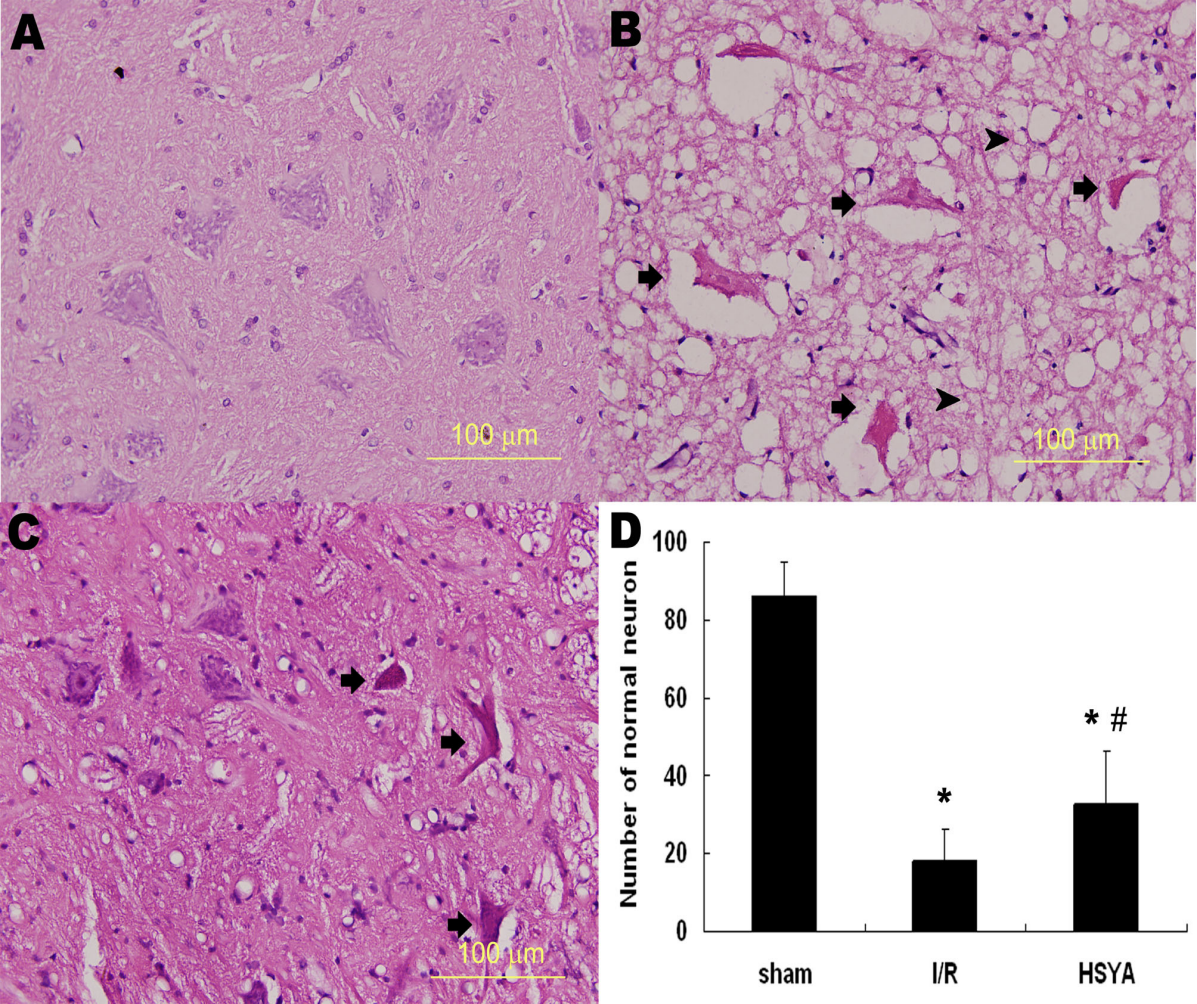
**Micrographs of hematoxylin and eosin staining (×400)**. (A) The micrographs from an animal with Tarlov score 4 in sham group showed no histopathology abnormity. (B) The image from an animal with Tarlov score 0 in I/R group. The arrows indicate ischemia neuron cells showing intensely eosinophilic cytoplasm, Nissl body loss, and pyknosis. The arrow heads indicate pronounced vacuolization in the anterior horn. (C) The micrographs from an animal with Tarlov score 2 in HSYA-treatment group revealed alleviated destruction after HSYA treatment. (D) The number of normal motor neurons in the anterior horn 48 h after reperfusion. * *P *< 0.05 versus sham group; ^# ^*P *< 0.05 versus I/R group.

Representative histopathological photographs of sections are shown in Figure 1. Representative image from an animal with Tarlov score 4 in sham group showed no signs of histopathological abnormalities (Figure 1A). In contrast, the image from an animal with Tarlov score 0 in I/R group exhibited necrotic changes with pronounced vacuolization, intensely eosinophilic cytoplasm, Nissl granule loss, and pyknosis (Figure 1B). HSYA treatment attenuated I/R-induced histopathological abnormalities. Representative images from HSYA group exhibited mild destruction with significantly more normal neurons (Figure 1C and Figure 1D).

### Biochemical analysis

As seen from Table 2, spinal cord MDA level of I/R group was significantly higher than that of sham group (p < 0.05). This increase in MDA content was counteracted by HSYA administration. As for SOD activity, a significant decrease was observed in I/R group when compared with that of sham group (p < 0.05). In animals treated with HSYA, SOD activity was markedly higher than that of I/R group.

**Table 2:** Effect of HSYA on MDA level and SOD activity (n = 5).

Experimental conditions	MDA (nm/gww)	SOD (U/mg protein)
Sham Group	51.87 ± 10.82	0.65 ± 0.04
I/R Group	135.32 ± 17.48*	0.44 ± 0.09*
HSYA Group	101.36 ± 19.06*^, #^	0.54 ± 0.08*^, #^

* p < 0.05, compared with sham group, ^# ^p < 0.05, compared with I/R group.

### TUNEL staining

**Figure 2:**
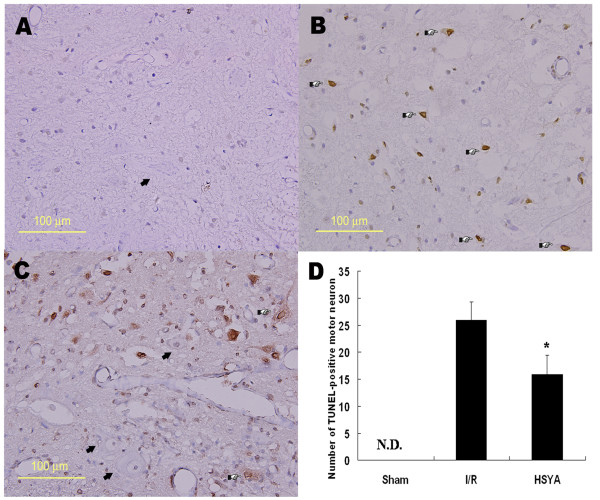
**Micrographs of TUNEL staining and quantification of apoptotic motor neurons 48 h after reperfusion (×400)**. (A) No TUNEL-positive neurons could be detected in sham group. (B) Many TUNEL-positive neuron with intense nucleus staining were visible in I/R group. (C) Only a small number of positively stained neurons were observed in the HSYA-treatment group. The arrows indicate normal motor neurons, and the arrowheads indicate TUNEL-positive motor neurons. (D) Quantitative analysis of the number of TUNEL-positive cells in the anterior horn of spinal cord of lumbar segments in three groups. * *P *< 0.05 versus I/R group. N.D. = not detected. The arrows indicate normal motor neurons, and the pointers indicate TUNEL-positive motor neurons.

The results of TUNEL staining are shown in Figure 2. While no TUNEL-positive neurons could be identified in sham group (Figure. 2A), numerous neurons with intensely stained nuclei could be detected in I/R group (Figure. 2B). HSYA administration reduced the number of apoptotic neurons (Figure. 2C). As seen from the quantitative analysis of neuronal apoptosis (Figure. 2D), the number of TUNEL-positive neurons decreased significantly after the treatment of HSYA, suggesting that HSYA may protect spinal cords from I/R-induced apoptosis.

## Discussion

This study demonstrates a considerable neuroprotective effect of HSYA against spinal cord ischemia/reperfusion injury in rabbits. Animals treated with HSYA exhibited improved neurological scores, reduced tissue destruction and oxidative stress, and decreased neuronal apoptosis.

Reactive oxygen species (ROS) induced damage has been accepted as an important contributor in post-ischemic cell injury and cell death in spinal cords [15]. ROS, consisting of oxygen free radicals and associated entities, are natural products in the metabolism of cells [16,17]. In pathological conditions, including I/R injuries, ROS are overproduced while antioxidative enzymes are inactivated and antioxidants over-consumed [6]. When the production of ROS overwhelms endogenous antioxidant defenses, excessive ROS would result in the self-perpetuating free radical reaction of lipid peroxidation, which could, subsequently, induce membrane damage, cell lysis, organelle dysfunction, and calcium dyshomeostasis. Since spinal cords are characterized by large lipid content and high oxygenation, they are especially susceptible to lipid peroxidation related cellular damage [18]. Therefore, salvaging the spinal cord from oxidative damage by using antioxidant therapies is an important area of continuing investigation in spinal cord I/R injuries. As a matter of fact, high-dose of methylprednisolone, which is considered the only widely accepted pharmacological therapy currently in use in human spinal cord injuries, confers neuroprotection through its ability to inhibit post-traumatic lipid peroxidation and inflammatory responses.

In traditional Chinese medicine, the flower of the safflower plant, *Carthamus tinctorius *L., has been extensively used in the treatment of cerebrovascular and cardiovascular diseases. The extracts of *Carthamus tinctorius L*. contain yellow and red pigments including hydroxysafflor yellow A (HSYA), safflor yellow B, safflomin A, safflomin C, as well as other chemicals. Among all these components, HSYA has been demonstrated as the most active chemicals. In 2003, Zhu et al. [19] first reported the neuroprotective effects of HSYA on cerebral ischemic injury in both *in vivo *and *in vitro *studies. Considering that oxidative stress is an important cause of tissue damage after I/R injury, Jin et al. [7] examined the possible antioxidative effect of HSYA and demonstrated that HSYA could scavenge hydroxyl radicals and inhibit lipid peroxidation *in vitro*. The antioxidative effect of HSYA, as proposed by Jin et al., may be attributed to its multiple phenolic hydroxyl groups. In accordance with Jin's study, Tian et al. [20,21] further demonstrated that HSYA protected the cortex mitochondrial against I/R injuries through scavenging of free radicals, reduction of lipid peroxidation, inhibition of Ca^2+ ^overload, and inhibition of the opening of mitochondrial permeability transition pores (mtPTP). Later, Wei's study of focal cerebral ischemia reperfusion injury showed that, compared with animals that received no pharmacological treatment, animals treated with HSYA revealed reduced MDA content, increased SOD activity and total antioxidative capability in the brain and serum [9]. This study provided direct *in vivo *evidence that HSYA confers neuroprotection through its antioxidative action. Moreover, as reported by Zhu [22] and Ye [23], the neuroprotective effect of HSYA might also be attributed to its inhibition of thrombosis formation and platelet aggregation, its regulation on PGI2/TXA2 (prostaglandin I2/thromboxane A2) ratio and blood rheological changes, as well as its suppression of inflammatory responses.

Although beneficial effects of HSYA have been demonstrated in I/R injuries of various organs, its effect on spinal cord I/R injuries has not been studied yet. Thus, we conducted the present study to investigate the possible neuroprotective effect of HSYA against spinal cord I/R injuries in rabbits. The dose of 10 mg/kg was chosen based on our prior preliminary study and related references which demonstrated remarkable protection of HSYA against brain I/R injury at the dose of 8 mg/kg in rats [9].

The hypothesis that HSYA could protect against spinal cord I/R injury was accepted because neurological evaluation after reperfusion revealed improved motor function of hinder limbs in animals that received HSYA treatment. However, neurological scores of animals in HSYA group were still lower than that of sham group, suggesting that HSYA alone may not be effective enough to completely restore motor functions. The beneficial effect of HSYA was also confirmed by histopathological study. While severe vacuolization of gray matter and degeneration of motor neurons were observed in the non-treated group, only mild tissue destruction with significant more normal motor neurons were detected in the HSYA-treated group.

To further answer the question of whether the protective effect of HSYA was related to its antioxidative efficiency, we performed the biochemical analysis to study the changes in MDA level and SOD activity in spinal cords. Lipid peroxidation induced by ROS is a primary cause of reperfusion injury in spinal cords when blood flow is restored, and the level of MDA, a relatively stable end product of lipid peroxidation, is considered as indirect evidence of reperfusion injury. In accordance with other researches, a significant increase in MDA content was observed in the spinal cord tissue of rabbits in I/R group [24]. HSYA significantly attenuated the increase of MDA in the tissue. We therefore concluded that the protective effect of HSYA in I/R-induced spinal cord injury may be partly related to its anti-lipid peroxidative properties. SOD, a protective enzyme that scavenges the superoxide radical by catalyzing its dismutation to hydrogen peroxide and oxygen, is the first line of defense against free radical generation. The overproduction of ROS can cause SOD consumption and depletion. In the current study, SOD activity of the spinal cord tissue was greatly decreased by I/R, and was restored by HSYA treatment. However, the precise mechanism of the effect of HSYA on SOD activity remains to be determined.

Remembering that apoptosis is responsible for delayed neuronal cell death after I/R injury [25], we further studied the effect of HSYA on neuronal apoptosis by TUNEL staining. Compared with control group, HSYA significantly reduced the number of TUNEL-positive cells in the anterior horn of spinal cords. This protective effect of HSYA against neuronal apoptosis may be related to its antioxidative efficiency because the major mechanism of I/R induced apoptosis is attributed to ROS release [26]. Recent studies have revealed that antioxidants attenuated ischemic neuronal apoptosis through Bcl-2 up-regulation and Bax down-regulation [27]. The exact mechanism of HSYA's anti-apoptotic effect will be explored in further studies.

## Conclusions

The results of this study indicate that the potent antioxidant HSYA could protect spinal cords from I/R injury by alleviating oxidative stress and reducing neuronal apoptosis. Neurological evaluation, histological examination, biochemical study, and TUNEL staining all revealed improved outcomes in animals treated with HSYA. Considering that I/R injury of spinal cords is a complicated biological process, we suppose that more efficacious therapies may be achieved by the combination of several pharmacological agents targeting at different pathological pathways. Therefore, in future researches, besides further elucidation of the protective effect of HSYA, we will also explore important mechanisms of spinal cord I/R injury and try to combine several pharmacological agents to find a more effective pharmacological strategy to protect against spinal cord I/R injury.

## Abbreviations

I/R: ischemia/reperfusion; HSYA: Hydroxysafflor Yellow A; MDA: malondialdehyde; SOD: superoxide dismutase; TUNEL: TdT-mediated dUTP nick end labeling; ROS: reactive oxygen species.

## Competing interests

The authors declare that they have no competing interests.

## Authors' contributions

SLQ participated in the design of the study, carried out the tissue assays and animal experiments, performed the statistical analysis, and wrote the manuscript. MS participated in the design of the study, carried out all in vivo studies, contributed to the manuscript preparation. ZY assisted in the animal experiments and helped to draft the manuscript. ZY assisted in the biochemical assays. ZLH assisted in the TUNEL staining. RPC assisted in the anesthesia of the animals. WYC contributed to evaluate the neurological status of the animals. FQY made contributions to the overall design of the study. MBA (corresponding author) made substantial contributions to the overall design of the study and directed the research. All authors read and approved the final version of the manuscript.
